# Lipoprotein cholesterol ratios and cardiovascular disease risk in US adults: a cross-sectional study

**DOI:** 10.3389/fnut.2025.1529223

**Published:** 2025-04-17

**Authors:** Xiuming Yang, Qiuyun Chen, Qingyu Zhang, Zongliang Yu

**Affiliations:** ^1^Department of Cardiology, Affiliated Kunshan Hospital of Jiangsu University, Kunshan, Jiangsu, China; ^2^Department of Cardiology, Gusu School, Nanjing Medical University, The First People's Hospital of Kunshan, Kunshan, Jiangsu, China

**Keywords:** lipoprotein ratio, cvd, lipid metabolism, NHANES, cardiovascular risk prediction

## Abstract

**Background:**

The ratio of non-high-density lipoprotein cholesterol to high-density lipoprotein cholesterol (NHHR) has been introduced as a novel indicator to evaluate lipid metabolism. The study explored the association between NHHR and cardiovascular disease (CVD).

**Methods:**

A cross-sectional study was achieved by utilizing data obtained from the NHANES (2003–2016). The association between NHHR and CVD was assessed by multivariate logistic regression analysis (LRA) and the restricted cubic spline (RCS) analysis. Also, interaction tests and subgroup analyses were employed to explore whether the associations differ by subgroups. Then, threshold analysis were conducted for interval delineation and detection of threshold effects with two-segment piecewise LR model.

**Results:**

A cohort of 11,471 individuals was involved. The results indicated that the linear relationship between NHHR and CVD was not significant (*P* for trend >0.05). The RCS analysis revealed a non-linear J-shaped association of NHHR with CVD risk. A two-segment LR model was established to assess the threshold effect of the NHHR. A log-likelihood ratio test (*P* < 0.001) suggested that the two-segment LR model exhibited better performances compared with the single-line LR model. Additionally, a tangent point of the NHHR occurred at 2.82, and the likelihood of CVD increased by 21% as the NHHR increased by one unit (OR = 1.21, 95% CI = 1.10–1.34).

**Conclusions:**

A J-shaped association was detected between NHHR and the prevalence of CVD, suggesting that NHHR could serve as a novel assessment marker for identifying high-risk CVD populations. However, further cohort studies are needed to confirm this finding.

## 1 Introduction

Cardiovascular disease (CVD) remains a significant global health threat. According to the Global Burden of Disease (GBD) Study 2019, the total number of CVD cases nearly doubled from 271 million in 1990 to 523 million in 2019 (1). Recent data from the American Heart Association (AHA) reveal that in 2021, CVD was responsible for ~19.91 million deaths worldwide, with an individual in the United States succumbing to CVD every 34 s on average (2). Furthermore, a recent European study forecasts that between 2025 and 2050, the prevalence of cardiovascular diseases will surge by 90%, with the number of cardiovascular-related deaths projected to reach 35.6 million by 2050 (3). These alarming statistics underscore the urgent need for enhanced global strategies and interventions to mitigate the impact of CVD.

Atherosclerosis is crucial in the development of CVD (4). Hyperlipidemia can impair arterial endothelial function and heighten the susceptibility to atherosclerosis. The association of CVD and lipid metabolism is intricate and intimate. Lipid metabolism disorder serves as one of CVD's risk factors and has been playing a crucial role in the pathophysiological processes. Exposure to low-density-lipoprotein cholesterol (LDL-C) and other mediators of cardiovascular risks in young adults raises the incidence of subclinical atherosclerosis and is related to elevated incidence of cardiovascular events in later life (5). Apart from high total cholesterol and low HDL-C, high LDL-C is a pivotal factor influencing both atherosclerosis and cardiovascular metabolism (6). The accumulation of cholesterol-rich residual particles in patients with hypertriglyceridemia can also cause atherosclerosis and trigger atherosclerotic cardiovascular disease (AsCVD) (7). It has been demonstrated that non-HDL-C can play the same crucial role as LDL-C in incidence prediction of atherosclerosis and CVD (8–12). The non-HDL-C comprises the total content of cholesterol found in very low-density lipoprotein (VLDL), intermediate-density lipoprotein (IDL), LDL-C and lipoprotein(a) (all atherogenic lipoproteins) (8, 13). Some international guidelines recommended non-HDL-C for the risk assessment of ASCVD (14, 15). On the contrary, HDL-C, which is characterized by good antioxidant and anti-atherosclerotic performances, was negatively related to the incidence of ASCVD (16, 17). The non-HDL-C, as an independent predictor of residual cardiovascular risk, may provide additional information with its ratio to HDL-C (NHHR) to enhance CVD risk stratification (18). As a novel composite lipid metric, NHHR integrates proatherogenic (non-HDL-C) and atheroprotective (HDL-C) components, offering a holistic approach to cardiovascular risk assessment (19). The NHHR includes both atherogenic and anti-atherogenic lipid markers, providing a better understanding of lipid health. It can effectively evaluate the severity of atherosclerosis and holds predictive value in metabolic disorders such as diabetes and kidney-related disease (20–23).

CVD has been recognized as a metabolic disorder associated with atherosclerosis. A European prospective study involving 46,786 participants has suggested that NHHR served as a better risk marker for coronary heart disease than single LDL-C in patients with type 2 diabetes mellitus (24). To date, the association of NHHR as a comprehensive indicator with CVD remains unclear in American adults. Herein, we hypothesized a significant association of NHHR with CVD. Afterwards, a thorough analysis was performed to explore the association of the NHHR with the risk of CVD among American adults based on data collected from the NHANES (2003–2016).

## 2 Materials and methods

### 2.1 Survey

The NHANES evaluates the health and nutritional status of the U.S. population. It collects questionnaire responses and biological samples from nationally representative groups annually, with findings reported every 2 years. The study protocol had been approved by the National Center for Health Statistics (NCHS), and all participants have signed informed consent.

### 2.2 Participants

**Figure 1:**
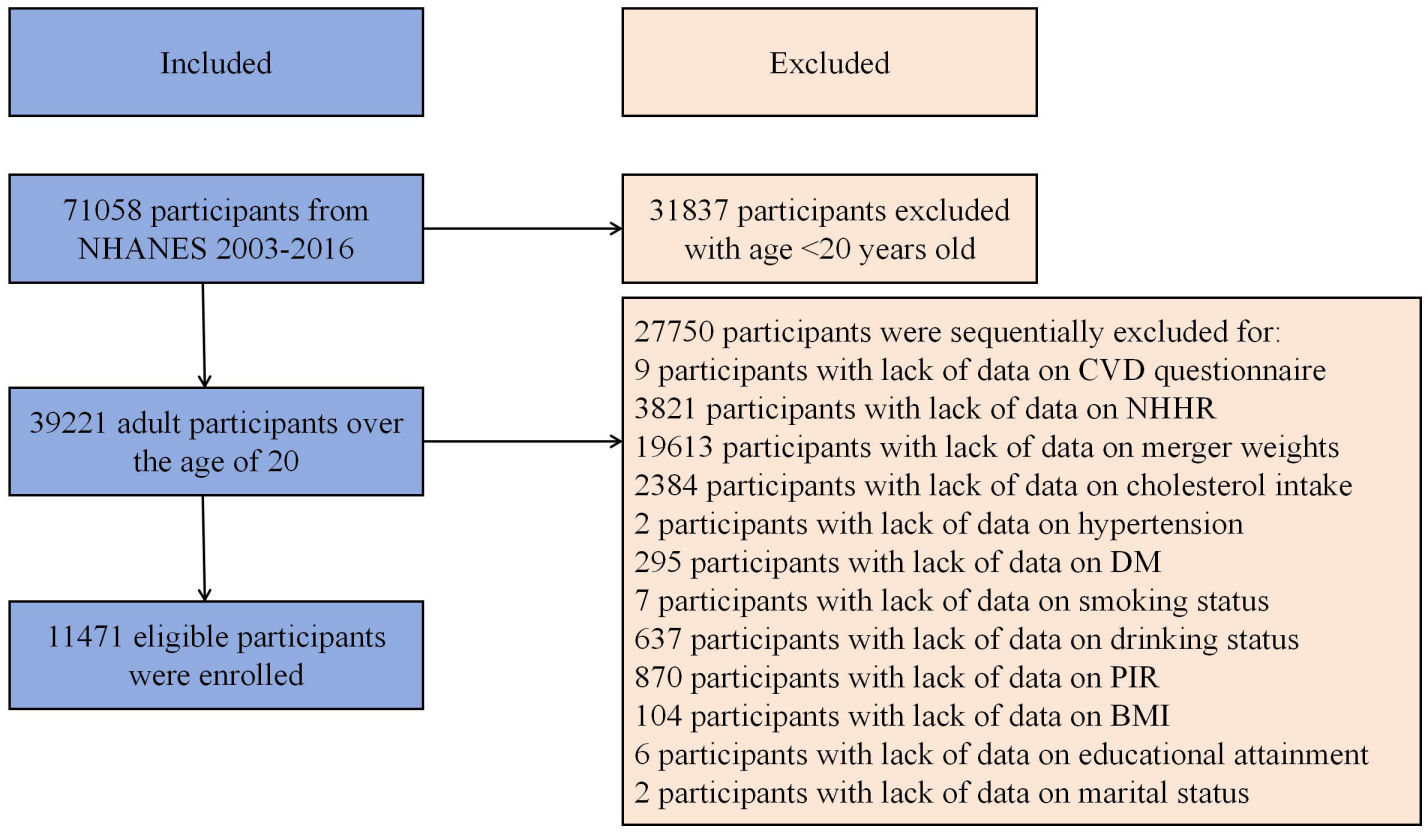
Flow diagram of study cohort selection.

This study used data extracted from the 2003–2016 US NHANES. Exclusion criteria: (1) Missing NHHR data, (2) Missing CVD data identified as missing data from MCQ160B to MCQ160F in medical conditions questionnaire, and (3) Absence of other covariate data. A total of 11,471 participants were involved (Figure 1). Detailed exclusion of participants with missing information of covariates were presented in Figure 1.

### 2.3 Exposure and outcomes

The NHHR serves as the exposure variable (25). The non-HDL-C level was determined on the basis of the levels of total cholesterol and HDL-C (26). The measurements of total cholesterol and HDL-C were substantially explained in the NHANES website (https://wwwn.cdc.gov/nchs/data/nhanes/public/2015/labmethods/TCHOL_I_MET.pdf). NHHR was calculated as the following formula: NHHR = (Total cholesterol – HDL-C)/HDL-C. The outcome variable was the CVD diagnosis, which relies on self-reported physician diagnoses obtained by a uniform standardized medical questionnaire survey. The question was “Has a doctor or other healthcare professional ever told you that you have coronary heart disease, angina, heart attack, stroke, or similar conditions?” Individuals who responded affirmatively to any of these conditions were regarded as CVD patients.

### 2.4 Covariates

This study incorporated abundant covariates related to NHHR and the risk of CVD. The variables included age, gender, race, marital status, educational level, smoking and drinking history, poverty income ratio (PIR), body mass index (BMI), dietary cholesterol intake, hypertension, and diabetes mellitus (DM). The serum biomarkers analyzed in this study included cholesterol and HDL-C (both in mmol/l). More details about the covariates are available in the Supplementary Table S1.

### 2.5 Statistical analysis

In this study, R software was utilized for statistical analysis, which included the specific sub-weight (WTSAF2YR) divided by 7 of NHANES samples to account for the complexities of multi-stage cluster sampling since 7 cycles of data were combined, following the guidelines by the CDC for sample weight calculation. Subjects were categorized based on NHHR quartiles. Continuous and categorical variables were investigated by using one-way ANOVA and chi-square tests, respectively, and described as mean ± standard deviation (SD) and frequencies and percentages, respectively. The baseline characteristics of NHHR were contrasted across four quartiles. A multivariate LR model comprising three models to accommodate confounding factors was developed to assess the relationship between NHHR and CVD risk. Specifically, Model 1 excluded covariates, Model 2 involved adjustment for the demographic factors mentioned above, and Model 3 included demographic factors, BMI, PIR, smoking and drinking history, hypertension, DM, dietary cholesterol intake and total cholesterol. RCS LR was employed to explore the nonlinear associations of NHHR and the risk of CVD. In cases where nonlinear relationships were observed, a two-segment piecewise LR model was established for interval delineation and detection of threshold effects. Subgroup analyses were also performed. *P* < 0.05 denoted statistical significance.

## 3 Results

### 3.1 Baseline features based on NHHR quantiles

Table 1 displays the baseline features of participants, categorized into quartiles based on NHHR. An aggregate of 11,471 participants representative for 167.6 million non-institutionalized American adults (mean age = 47.73 years, SD of age = 0.27 years) were involved, among which 48.47% were male and 51.53% were female. Noticeable differences were detected among groups regarding demographic factors, hypertension, PIR, DM, BMI, dietary cholesterol intake, total cholesterol levels, and HDL-C (*P* < 0.05). To be more specific, the proportions of male and non-Hispanic White were higher in groups with higher NHHR. Moreover, individuals with elevated NHHR exhibited lower level of education and income, as well as higher rates of smoking and alcohol consumption. Elevated NHHR was also related to higher BMI and total cholesterol, higher prevalence of hypertension and diabetes mellitus as well as low HDL-C (*P* < 0.001).

**Table 1:** Weighted characteristics of the study population according to the quartiles of NHHR.

**Characteristics**	**Total**	**Q1 ( ≤ 1.92)**	**Q2 (1.93 to ≤ 2.62)**	**Q3 (2.63 to ≤ 3.55)**	**Q4 (> 3.55)**	***P*-value**
Number	11,471	2,815	2,877	2,847	2,932	
Age (years)	47.73 (0.27)	47.12 (0.49)	48.33 (0.45)	48.23 (0.40)	47.23 (0.37)	< 0.001
**Sex (%)**						
Male	5,640 (48.47)	963 (31.70)	1,237 (42.25)	1,537 (53.48)	1,903 (65.81)	< 0.001
Female	5,831 (51.53)	1,852 (68.30)	1,640 (57.75)	1,310 (46.52)	1,029 (34.19)	
**Race (%)**						
Non-Hispanic White	5,665 (71.92)	1,365 (70.38)	1,395 (71.40)	1,422 (73.37)	1,483 (72.47)	< 0.001
Non-Hispanic Black	2,167 (10.35)	688 (13.56)	612 (11.43)	475 (9.14)	392 (7.38)	
Mexican American	1,796 (7.38)	312 (5.45)	414 (6.78)	496 (7.92)	574 (9.28)	
Other Race	1843 (10.36)	450 (10.61)	456 (10.38)	454 (9.57)	483 (10.87)	
**Educational attainment (%)**						
High school or less	5,327 (38.45)	1,096 (31.83)	1,278 (35.79)	1,403 (40.90)	1,550 (45.01)	< 0.001
More than high school	6,144 (61.55)	1,719 (68.17)	1,599 (64.21)	1,444 (59.10)	1,382 (54.99)	
**Marital status (%)**						
Married or living with partner	7,074 (65.68)	1,554 (61.08)	1,714 (63.71)	1,845 (68.36)	1,961 (69.41)	< 0.001
Living alone	4,397 (34.32)	1,261 (38.92)	1,163 (36.29)	1,002 (31.64)	971 (30.59)	
**PIR (%)**						
Low	2181 (12.78)	478 (12.18)	542 (12.63)	529 (11.42)	632 (14.83)	< 0.001
Middle	6,232 (50.71)	1,483 (47.65)	1,535 (50.07)	1,617 (54.63)	1,597 (50.43)	
High	3,058 (36.52)	854 (40.18)	800 (37.30)	701 (33.95)	703 (34.74)	
**Smoking status (%)**						
Never	6,194 (53.76)	1,669 (58.54)	1,592 (55.84)	1,523 (52.56)	1,410 (48.28)	< 0.001
Now	2,242 (20.09)	445 (16.40)	528 (18.40)	536 (19.81)	733 (25.60)	
Former	3,035 (26.15)	701 (25.07)	757 (25.76)	788 (27.63)	789 (26.12)	
**Drinking status (%)**						
Never	1,530 (10.93)	395 (11.49)	411 (11.46)	384 (10.87)	340 (9.95)	< 0.001
Mild	3,918 (36.70)	951 (35.55)	1,000 (37.09)	1,004 (38.82)	963 (35.35)	
Moderate	1,666 (16.88)	522 (22.16)	424 (17.35)	345 (13.44)	375 (14.73)	
Heavy	2,123 (19.46)	524 (19.52)	498 (18.88)	514 (18.96)	587 (20.47)	
Former	2,234 (16.02)	423 (11.29)	544 (15.22)	600 (17.92)	667 (19.49)	
**Hypertension (%)**						
No	6,462 (61.59)	1,692 (67.38)	1,623 (62.68)	1,561 (59.65)	1,586 (56.85)	< 0.001
Yes	5,009 (38.41)	1,123 (32.62)	1,254 (37.32)	1,286 (40.35)	1,346 (43.15)	
**DM (%)**						
No	9,176 (84.94)	2,326 (88.00)	2,350 (85.81)	2,264 (84.66)	2,236 (81.41)	< 0.001
Yes	2,295 (15.06)	489 (12.00)	527 (14.19)	583 (15.34)	696 (18.59)	
**CVD (%)**						
No	10,125 (90.74)	2,456 (90.04)	2,534 (90.36)	2,536 (91.49)	2,599 (91.03)	0.392
Yes	1,346 (9.26)	359 (9.96)	343 (9.64)	311 (8.51)	333 (8.97)	
BMI (kg/m^2^)	29.00 (0.10)	26.01 (0.14)	28.51 (0.16)	30.16 (0.20)	31.22 (0.16)	< 0.001
Dietary cholesterol intake (mg)	286.85 (2.43)	264.31 (4.16)	280.17 (4.31)	290.28 (4.68)	311.76 (4.60)	< 0.001
Total cholesterol (mmol/l)	5.04 (0.01)	4.47 (0.02)	4.80 (0.02)	5.13 (0.02)	5.75 (0.02)	< 0.001
Low risk (2.07 to ≤ 4.50)	3,845 (32.49)	108 (3.29)	441 (14.05)	1,062 (35.52)	2,234 (75.71)	< 0.001
Medium risk (4.50 to ≤ 5.38)	3,833 (33.88)	557(19.19)	1,249 (44.21)	1,376 (49.19)	651 (22.89)	
High risk (5.38 to ≤ 16.81)	3,793 (33.64)	2,150 (77.52)	1,187 (41.74)	409 (15.28)	47 (1.40)	
HDL-C (mmol/l)	1.40 (0.01)	1.83 (0.01)	1.48 (0.01)	1.27 (0.01)	1.04 (0.01)	< 0.001
**HDL-C tertile**						
High risk (0.16 to ≤ 1.16)	3,855 (32.70)	1,588 (54.70)	1,199 (40.33)	750 (25.84)	318 (10.73)	< 0.001
Medium risk (1.16 to ≤ 1.5)	3,804 (33.86)	820 (30.41)	999 (36.60)	1,114 (38.27)	871 (30.16)	
Low risk (1.5 to ≤ 5.84)	3,812 (33.45)	407 (14.89)	679 (23.07)	983 (35.88)	1,743 (59.11)	

Values are weighted means (standardized errors) or number of participants (weighted percentages) unless otherwise indicated.

### 3.2 Association of NHHR with CVD

The association of NHHR with CVD was analyzed by a multivariate LRA and the results are presented in Table 2. According to Model 3 (fully adjusted), every extra unit of NHHR corresponds to a non-significant OR of 1.10 (95% CI: 1.00–1.21, *P* = 0.051) for the prevalence of CVD. Additionally, NHHR was categorized into stratified variables for further analysis. The prevalence of CVD in groups Q2, Q3, and Q4 was 0.89 (95% CI: 0.70–1.14), 0.81 (95% CI: 0.62–1.06), and 1.07 (95% CI: 0.82–1.41), respectively, compared with group Q1. The tendencies observed with the *P* value exceeding 0.05 indicate that there could be a non-linear association of NHHR with CVD.

**Table 2:** Weighted multivariate logistic regression analysis of NHHR and CVD.

**Characteristic**	**Model 1 OR (95% CI), *P* value**	**Model 2 OR (95% CI), *P* value**	**Model 3 OR (95% CI), *P* value**
NHHR (continuous)	0.99 (0.93, 1.05), 0.713	1.03 (0.96, 1.11), 0.361	1.10 (1.00, 1.21), 0.051
**NHHR (categorical)**			
Q1 ( ≤ 1.92)	Reference	Reference	Reference
Q2 (1.93 to ≤ 2.62)	0.96 (0.77, 1.21), 0.749	0.92 (0.71, 1.17), 0.485	0.89 (0.70, 1.14), 0.363
Q3 (2.63 to ≤ 3.55)	0.84 (0.68, 1.05), 0.118	0.81 (0.64, 1.03), 0.088	0.81 (0.62, 1.06), 0.120
Q4 (> 3.55)	0.89 (0.72, 1.09), 0.266	0.96 (0.77, 1.22), 0.761	1.07 (0.82, 1.41), 0.602
*P* for trend	0.153	0.557	0.891

Model 1: unadjusted; Model 2: adjusted for age, sex, race, educational attainment, and marital status; Model 3: adjusted for age, sex, race, education attainment, marital status, BMI, PIR, smoking status, drinking status, hypertension, DM, dietary cholesterol intake and total cholesterol.

### 3.3 Nonlinear association of NHHR with CVD

**Figure 2:**
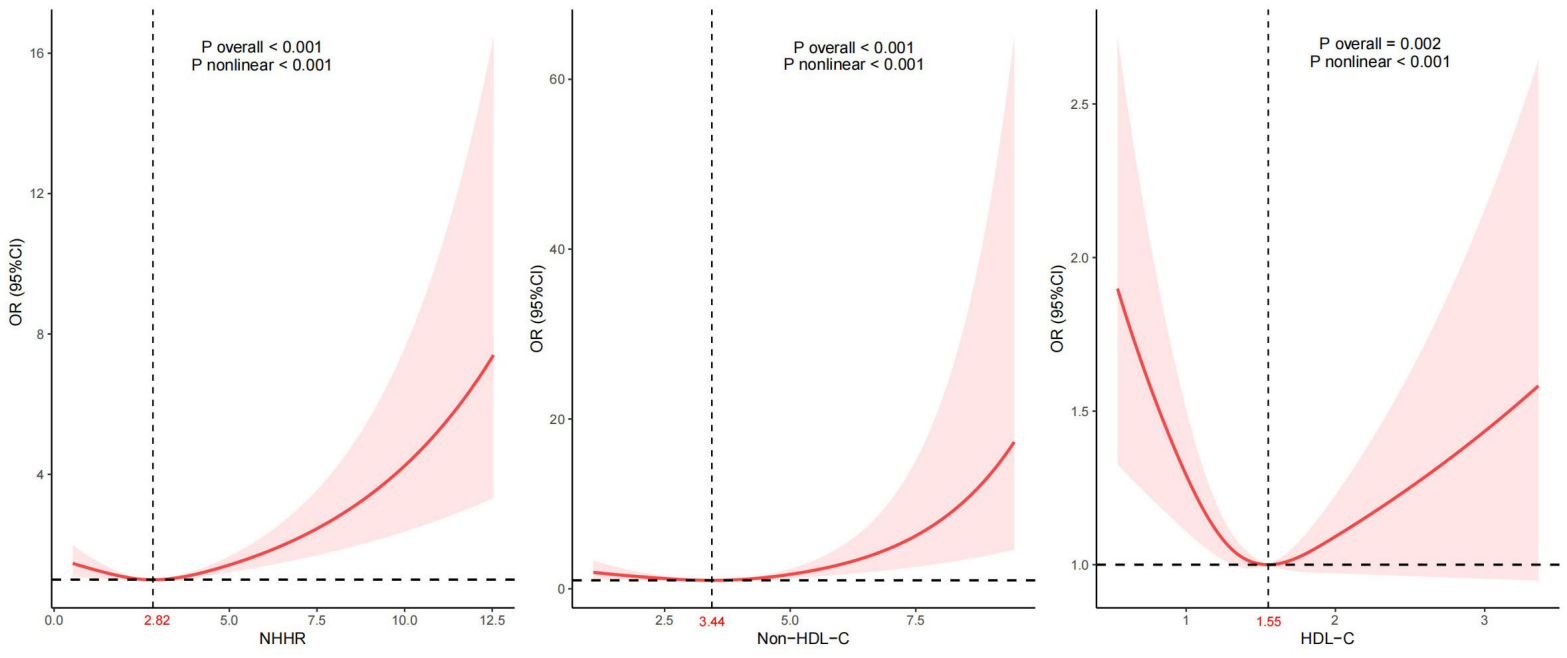
Association between NHHR and CVD. Adjustment factors included age, sex, race, education attainment, marital status, BMI, PIR, smoking status, drinking status, hypertension, DM, dietary cholesterol intake, and total cholesterol.

Given that the NHHR is a continuous variable, it is essential to examine potential nonlinear associations. Analysis by using a RCS regression model indicated a J-shaped relationship characterized by a curve that reached its nadir at an inflection point of 2.82 in the NHHR. We also found a non-linear relationship between HDL-C, non-HDL-C, and CVD (Figure 2). Life's Crucial 9 (LC9) is an emerging cardiovascular health scoring system that incorporates Life's Essential 8 alongside mental health factors. We additionally adjusted for LC9 in our sensitivity analyses to account for the influence of dietary and lifestyle factors on our findings. Despite this adjustment, we observed a persistent nonlinear relationship between NHHR and CVD with the threshold shifting rightward by 0.24 compared to the previously identified threshold (Supplementary Figure S1).

**Figure 3:**
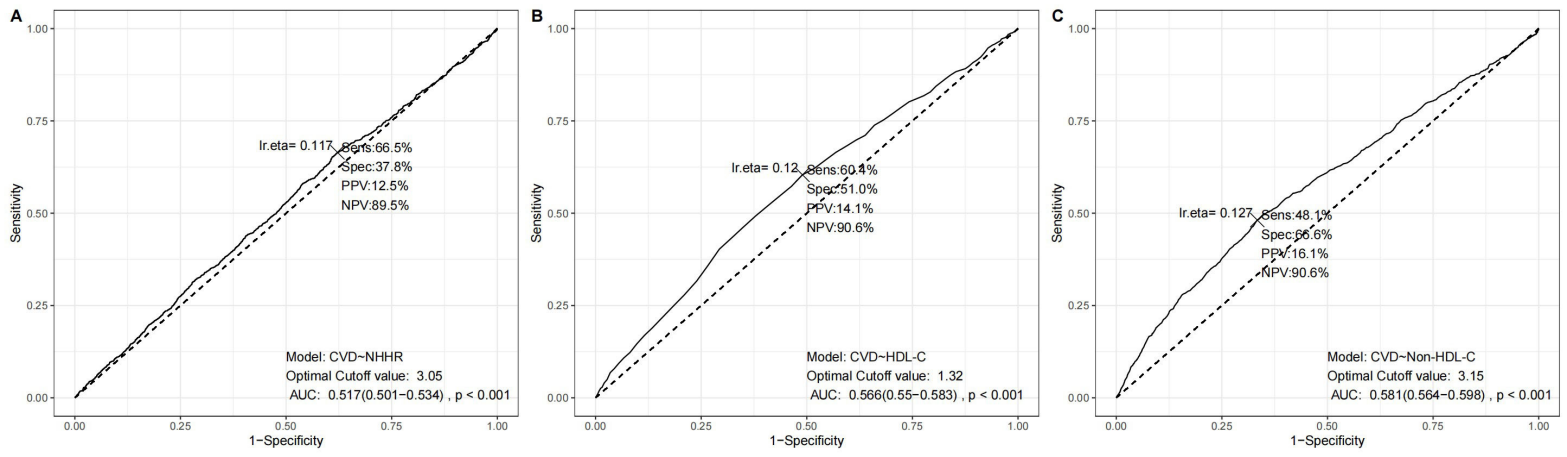
ROC results using different lipid parameters. **(A)** NHHR model, **(B)** HDL-C model, and **(C)** Non-HDL-C model. Each panel shows sensitivity, specificity, positive predictive value (PPV), negative predictive value (NPV), area under the curve (AUC), and statistical significance at the optimal cutoff value.

Moreover, we conducted receiver operation characteristics curvere regarding NHHR, HDL-C, and non-HDL-C, and the results was illustrated in Figure 3 with sensitivity and specificity. The true positive rate (sensitivity) for NHHR, HDL-C, and non-HDL-C in the best thresholds were 66.5%, 60.4%, and 48.1%, in which NHHR possess the greatest sensitvity. As presented in Supplementary Table S2, 1,411 and 3,183 participants would be reclassified to lower risk categories than the use of HDL-C and non-HDL-C, indicating the relatively high sensitivity of NHHR as an index of cardiovascular disease. This means that the NHHR is able to identify cardiovascular disease in the early stage, which could help in prompting clinicians to take more proactive steps in prevention and intervention. We also conducted additional analyses comparing NHHR's predictive performance with apoB and LDL-C in adjusted models. While the AUC values for NHHR (0.850), apoB (0.849), and LDL-C (0.850) demonstrated comparable discriminative capacity for CVD risk prediction (Supplementary Figure S2).

### 3.4 Threshold effect and subgroup analysis

The two-piecewise LR model was employed to assess the threshold effect of NHHR on the risk of CVD, wherein 2.82 was identified as the inflection point. Beyond this threshold, NHHR exhibited a positive association with CVD (OR = 1.21, 95% CI 1.10–1.3). The significant change was detected at the breakpoint (*P* < 0.001). For specific details, please refer to Table 3.

**Table 3:** Threshold effect analysis of the association of NHHR with CVD.

**NHHR**	**Adjust OR (95% CI)**	***P* value**
< 2.82	0.84 (0.70, 1.02)	0.072
≥2.82	1.21 (1.10, 1.34)	< 0.001
Log-likelihood ratio test		< 0.001

The threshold effect analysis was adjusted for age, sex, race, education attainment, marital status, BMI, PIR, smoking status, drinking status, hypertension, DM, dietary cholesterol intake, and total cholesterol.

**Figure 4:**
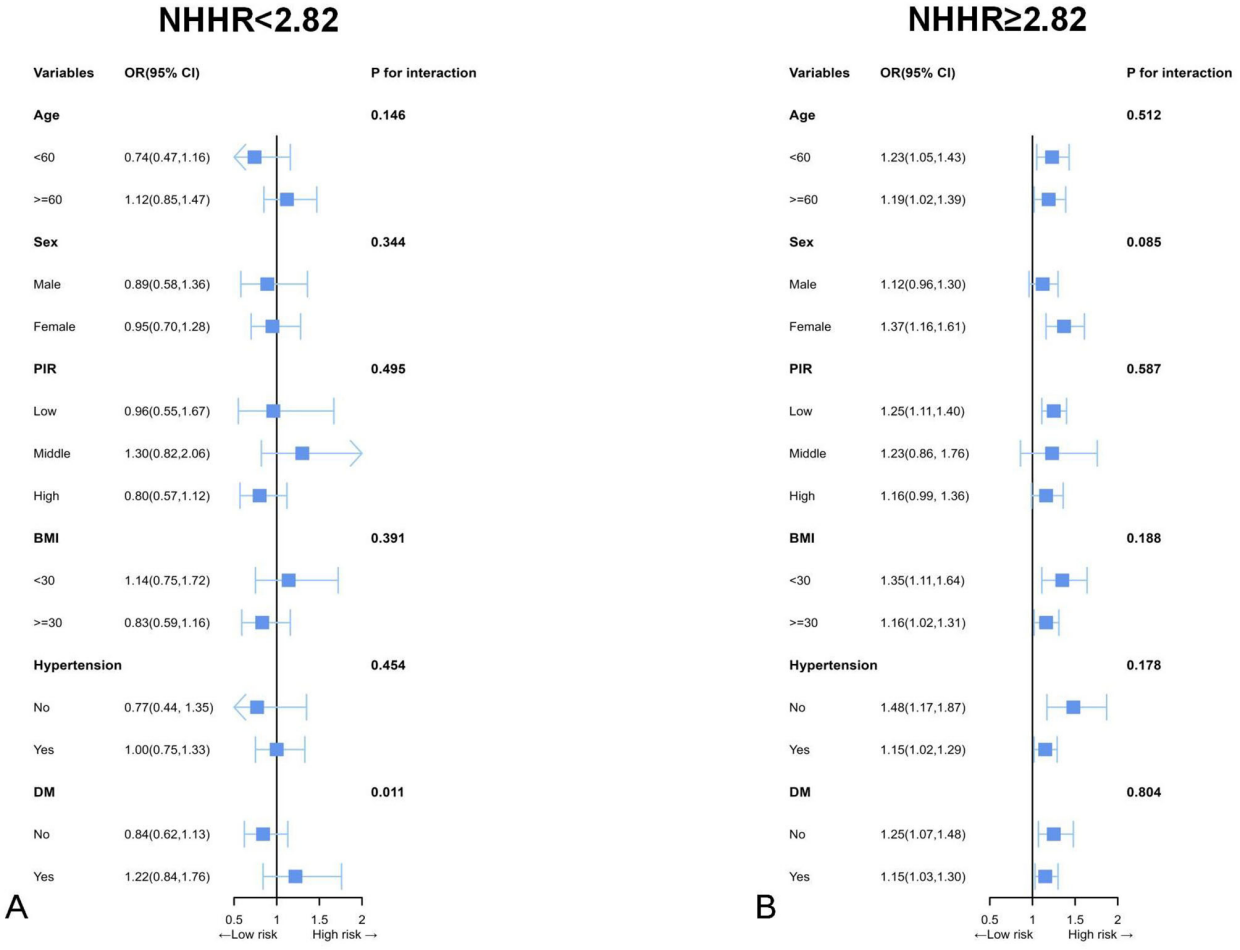
Subgroup analyses by possible effect modifiers for the relationship between NHHR and CVD divided by 2.82. **(A)** NHHR < 2.82 (*n* = 6,458); **(B)** NHHR ≥ 2.82 (*n* = 5,983). Each subgroup analysis adjusted for age, sex, race, education attainment, marital status, BMI, PIR, smoking status, drinking status, hypertension, DM, dietary cholesterol intake, and total cholesterol. Except for the stratifying variable.

As shown in Figure 4, subgroup analyses considering various demographic factors were employed for robustness evaluation of the association of NHHR and CVD for different populations. Subgroup analyses considering BMI, age, hypertension, gender, and diabetes mellitus were performed to assess the specific associations of NHHR with CVD for various populations. Herein, the population was partitioned into according to the inflection point of NHHR and the continuity association of NHHR and CVD in the different subgroups was thoroughly analyzed. The results indicated that a significant interaction between NHHR and CVD was observed exclusively in the DM subgroup when NHHR fell below 2.82. When NHHR exceeds 2.82, the significant associations between higher NHHR and higher CVD risk were identified in various subgroups except for male adults and adults with middle or higher PIR. Moreover, no interaction effects were observed, indicating the stability of the association among plenty of subpopulations.

## 4 Discussion

This cross-sectional analysis is essentially the first study of the association between NHHR and CVD risk in American adults. The results indicated a J-shaped association of NHHR and CVD. Moreover, an inflection point (2.82) was revealed by threshold effect analysis. This finding suggests that higher levels of NHHR are associated with an increased risk of CVD. When NHHR > 2.82, NHHR and CVD had a significant association in American adults.

In this study, we firstly compared the characteristics among participants across 4 quartiles of NHHR, and the results revealed the significant differences in lifestyles and NHHR. For instance, participants in the highest NHHR quartile had the highest BMI, and tended to current smoker and heavy drinker, consistent with previous lipid metabolism biomarkers (27, 28). And the results indicated the ability of NHHR serving as a indicator of lipid metabolism from another aspect. Then we conducted multivariate LR models to explore the associations between NHHR and CVD risk, but no significant associations in LR models were observed. Nevertheless, we additionally employed threshold effect analysis since total cholesterol and HDL-C were recommended to maintain at a medium level, and the results revealed the significant association when NHHR was more than 2.82. Meanwhile, the subgroup analyses further indicated the significant associations of NHHR and CVD prevalence in various subpopulations when NHHR reached 2.82. Nevertheless, the associations were not significant in male adults and participants with medium family income, which deserves in-depth explorations in subsequent studies. After consulting the literature, we found that the gender differences between lipid metabolism and CVD is controversial. For instance, a study involving 21 countries found that lipid markers and depression are more strongly associated with CVD risk in men than in women (29). Nevertheless, another study of ~2 million young adults demonstrated an association between the number of abnormal lipid profiles and incident CVD in both men and women, and no gender differences were found except for the associations of abnormal lipid profiles and incident myocardial infarction, which were more pronounced in men than women (30).

This study serves as an initial exploration of the direct association of NHHR and CVD. Our study is the first to demonstrate a non-linear association between NHHR and CVD risk, identifying a critical threshold of 2.82. When NHHR levels exceed this threshold, there is a significant increase in cardiovascular prevalence, which underscores the importance of early diagnosis and intervention in the management of cardiovascular diseases. The NHHR reflects the equilibrium of HDL-cholesterol and non-HDL-cholesterol, which play distinct roles in human body. Elevated NHHR could indicate disruptions in lipid metabolism. Previous studies have established dyslipidemia as a significant risk factor for CVD (31, 32). These findings align with our results demonstrating that this dyslipidemia is associated with an increased CVD risk. Elevated NHHR may be associated with decreased HDL levels, and may serve as a novel indicator of CVD. The Framingham Heart Study revealed a negative association of CVD and HDL-C (33) for the first time. Since then, it has been consistently held that HDL-C is negatively related to the risk of CVD, serving as a crucial component against CVD (34, 35). HDL performs a critical function in shielding against oxidative stress, particularly by inhibiting LDL from oxidative damage caused by ROS (36). This antioxidative role of HDL is partially mediated via the action of paraoxonase1 (PON1). PON1 activity aids in inhibiting formation of foam cells, thereby reducing the risk of atherosclerosis (37). Extensive research underscores the positive roles of HDL and PON1 in prevention of both atherosclerosis and CVD (36). Reduced HDL-C levels impair the lipoprotein's cholesterol efflux capacity while compromising its anti-inflammatory and antioxidant properties, as well as endothelial protective functions (38). This dual impairment contributes to elevated CVD risk. Non-HDL-C constitutes a significant component of NHHR, and a higher NHHR indicates an elevated level of non-HDL-C relative to HDL-C. Non-HDL-C represents the total cholesterol content of atherogenic lipoproteins that contain apolipoprotein B (ApoB), comprising very low-density lipoprotein (VLDL), intermediate-density lipoprotein (IDL), low-density lipoprotein (LDL), chylomicron remnants, and lipoprotein(a) [Lp(a)]. This composite measure of all atherogenic particles has emerged as a critical predictor of CVD risk (39). Its prognostic significance demonstrates enhanced predictive value particularly in younger populations and among individuals with well-controlled LDL-C levels (40). Prospective cohort studies have demonstrated that non-HDL-C constitutes an independent risk factor for ASCVD (41). Compared to LDL-C alone, non-HDL-C provides a more accurate assessment of atherosclerotic burden and demonstrates superior prognostic value for cardiovascular events (42, 43). Numerous studies indicate that high non-HDL-C levels are correlated with high risk of atherosclerosis and CVD (12, 44–46). In a study involving 21,448 participants from the EPIC cohort, patients with non-HDL-C levels > 130 mg/dL exhibited a hazard ratio (HR) of 1.84 for coronary heart diseases (95% CI = 1.12–3.04) (47). Elevated levels of non-HDL-C may increase the risk of CVD through several mechanisms. The lipoproteins in non-HDL-C, which contain ApoB, as well as remnants of VLDL and LDL particles, can freely cross the endothelial barrier and easily accumulate in the arterial wall, thereby triggering inflammatory response (48, 49). Additionally, oxidized low-density lipoprotein (ox-LDL) and LDL can bind to proteoglycans in the extracellular matrix of the vascular endothelium, leading to foam cell formation and exacerbating the inflammatory state within the arteries by recruiting circulating monocyte (50, 51). The oxidation of triglyceride-rich lipoproteins (TRLs) and the formation of residual particles can result in more pronounced inflammatory responses, which not only promote the progression of atherosclerosis but may also lead to plaque instability, thereby increasing the risk of cardiovascular events (52). When the NHHR exceeds 2.82, the risk of CVD significantly increases, which may be related to the cumulative effects of inflammation and vascular damage. Alyaydin et al. (53) found that elevated levels of residual cholesterol are associated with increased interleukin-6 levels (*p* = 0.025), indicating that residual cholesterol possesses pro-inflammatory properties. Furthermore, Li et al.'s (54) research emphasizes the relationship between non-HDL-C and inflammation, suggesting that an increase in non-HDL-C may serve as a marker of insulin resistance, which is itself a significant promoter of inflammation. The levels of non-HDL-C are elevated relative to HDL-C levels, suggesting a dominance of pro-atherosclerotic lipoprotein particle. This imbalance may lead to an increased risk of atherosclerosis and subsequently elevate the risk of CVD.

The J-shaped association between NHHR and CVD risk may arise from the interplay of lipid metabolic imbalance, inflammatory responses, and immunometabolic dysregulation. At lower NHHR levels (< 2.82), non-HDL-C particles infiltrate the vascular intima, inducing monocyte differentiation into macrophages and foam cell formation, while activating inflammatory pathways and oxidative stress, progressively triggering endothelial dysfunction and early plaque formation (55).During this phase, HDL-C exerts predominant protective effects by maintaining cholesterol efflux through reverse cholesterol transport (RCT) and counteracting inflammation, thereby mitigating the atherogenic effects of non-HDL-C (56). Near the threshold (NHHR is close to 2.82), the metabolic balance between non-HDL-C and HDL-C reaches a critical point. Prolonged exposure to elevated non-HDL-C drives oxidative modification of LDL (ox LDL) and cholesterol crystal deposition, activating the NLRP3 inflammasome and promoting IL-1β/IL-18 secretion, which amplifies vascular inflammation (57, 58). As NHHR levels increase, chronic inflammation induces glycation of apolipoprotein A1 (apoA1) in HDL-C, replacing functional components with pro-inflammatory mediators (e.g., SAA1, apo CIII), transforming HDL-C into a pro-atherogenic particle (59).Cumulative LDL-C exposure (quantified as LDL-C burden, LCB) correlates positively with CVD risk (60), activating the TLR4/NF-κB axis and recruiting neutrophils via CXCL1/CXCR2 signaling, further destabilizing plaques (6, 57, 61, 62).An increase in NHHR may contribute to metabolic disorders. In patients with cholestasis, the formation of Lp-X not only exacerbates lipid metabolism disorders (63), but also leads to electrolyte imbalances, further impairing vascular function. Additionally, the deposition of bilirubin and bile acids may worsen vascular endothelial damage by inducing oxidative stress and promoting the release of inflammatory factors (such as IL-6 and TNF-α), thereby indirectly disrupting cardiovascular homeostasis (64, 65).

NHHR, emerging as a novel indicator in lipid management, has been widely recognized for its close relation with various metabolic diseases (21–23, 66, 67). In populations with type 2 diabetes, the NHHR has demonstrated superior effectiveness in predicting cardiovascular risk compared to non-HDL-C or HDL-C (68). This study expands the population scope and further compares the correlation between NHHR and CVD risk in the general adult population of the United States, highlighting its superiority over single indicators such as LDL-C or non-HDL-C. You et al. (69) demonstrated that high NHHR was related to high incidence of acute coronary syndrome. Mao et al. (70) reported that NHHR was independently associated with adverse cardiovascular events and coronary artery lesions. Liu et al. (71) found that in patients with acute coronary syndrome (ACS) undergoing percutaneous coronary intervention (PCI), the NHHR is associated with the progression of coronary artery lesions. This study elucidates the nonlinear relationship between NHHR and CVD and establishes a threshold of 2.82, further emphasizing the clinical applicability of NHHR. Yu et al. (72) conducted a longitudinal cohort study revealing a non-linear association of NHHR and CVD-induced mortality with or at risk of diabetes, with a threshold effect of 2.83. In this study, a J-shaped association of NHHR and CVD was developed among the general population. Furthermore, a threshold effect analysis revealed that the inflection point was 2.82. Exceeding this threshold, the risk of CVD increased significantly. The consistent performance of NHHR across multiple validation cohorts, substantiates its clinical utility for CVD risk. Two studies also found the J-shaped association between NHHR and other outcomes shown as sarcopenia risk in individuals with cancer and prognosis in cancer survivors (73, 74). Moreover, the J-shaped association of lipid levels and all-cause and cause-specific mortality were substantially explored (75, 76). In fact, the J-shaped association of lipid markers are common since slight increase of LDL-C and decrease of HDL-C within the normal range would not significantly increase the risk of adverse outcomes. However, when it exceeds a threshold, the occurrence of hyperlipidemia would elevate oxidative stress (77) and promote chronic inflammation (78) and then lead to higher hypertension and CVD risk. Furthermore, multiple studies have illustrated the crucial role of lowering LDL-C and reducing hyperlipidemia risk in CVD management for primary prevention (79–81), indicating the potential utilization of NHHR as a novel biomarker in CVD prevention.

This study expands the understanding and application of lipid ratios by initially exploring and demonstrating a nonlinear relationship of NHHR and CVD, which holds significant implications for preventing and managing CVD from a lipid management perspective. In clinical practice, it is essential to prioritize the monitoring and management of NHHR, particularly when NHHR approaches or exceeds the established threshold, in order to implement proactive interventions aimed at reducing CVD risk. Moreover, this study also suggests that slight increase of NHHR would not elevate the CVD risk, reducing the excessive concerns of the public about the NHHR increase. Nevertheless, the role of NHHR as a target for lipid management and CVD prophylaxis and treatment shall be further clarified. This cross-sectional study, based on NHANES data, highlights the significance of integrating NHHR into a comprehensive risk assessment framework of CVD, rather than using it as a standalone metric. The findings suggest that NHHR may assist clinicians in developing personalized health interventions, particularly in modifying diet and lifestyle for high-risk individuals. Although lipid ratios are useful in population analyses, they should be employed cautiously and in conjunction with other diagnostic tools and biomarkers to provide a more thorough evaluation of cardiovascular and metabolic risk. It is crucial to acknowledge the limitations that, while cut-off points can yield clinically relevant information, employing quantiles within the cohort or population may be more logical for comparing ratios.

The clinical utility of NHHR extends beyond risk prediction to actionable prevention strategies. First, integrating NHHR into established frameworks, such as the ASCVD risk score or lipid management guidelines, could improve risk stratification. For instance, NHHR may serve as an additional marker for high-risk individuals to guide therapeutic decisions. A threshold of NHHR >2.82 should prompt intensified interventions, such as lifestyle modifications or pharmacotherapy. Second, automating NHHR calculation within electronic health records would enable real-time risk stratification without incurring additional costs. Third, prospective validation across diverse populations and clinical settings is essential to confirm its prognostic generalizability. Additionally, decision support tools could link NHHR levels to therapy escalation protocols, further enhancing its clinical applicability. Notably, NHHR's reliance on routine lipid measures makes it particularly valuable in resource-limited regions, helping bridge gaps in equitable risk assessment. By connecting risk assessment with therapeutic action, NHHR has the potential to reduce the global CVD burden through precision prevention.

### 4.1 Advantages and limitations

Advantages: Data was retrieved from the NHANES, which features a substantial sample size. Effective control for potential confounding factors was incorporated, thereby bolstering result reliability. Additionally, subgroup analysis and RCS analysis were utilized to explore the nonlinear association and evaluate the consistency of results across different populations.

Limitations: First, the cross-sectional design of the NHANES study precludes causal inference between NHHR and CVD due to the lack of temporality in exposure-outcome assessment. Second, cardiovascular disease diagnoses primarily relied on self-reported data, which may introduce recall bias, especially for asymptomatic conditions such as silent myocardial infarction. Future studies should integrate objective diagnostic tools including electrocardiography (ECG), coronary computed tomography angiography (CTA), or cardiac biomarkers such as troponin to validate these outcomes. Third, despite adjusting for multiple confounders, residual confounding from unmeasured factors such as genetic predisposition, socioeconomic status, or environmental exposures could persist. Lastly, the use of data spanning 2003 to 2016 may introduce temporal bias, as advancements in diagnostic criteria or healthcare practices during this period might influence the observed associations. Therefore, the clinical applicability of NHHR should be further verified using updated datasets.

## 5 Conclusions

In this study of American adults, a potential J-shaped association between NHHR and CVD was identified, with an optimal NHHR level of 2.82. Exceeding this threshold, higher NHHR may elevate the risk of CVD. This finding offers new insights into lipid management for CVD, although further cohort studies are necessary to validate these results.

## Data Availability

The datasets presented in this study can be found in online repositories. The names of the repository/repositories and accession number(s) can be found below: https://www.cdc.gov/nchs/nhanes.

## Supplementary material

Supplementary Figure S1 RCS analysis of the association between NHHR and CVD after full adjustment, including LC9 as an additional.

Supplementary Figure S2 ROC results. Comparison of NHHR with traditional lipid markers (LDL-C and apo B).

Supplementary Table S1 The detail definition and classification of covariates.

Supplementary Table S2 Best threshold-based risk reclassification for NHHR, HDL-C, and non-HDL-C.
