# Supplementary figures and images for: Lipoprotein cholesterol ratios and cardiovascular disease risk in US adults: a cross-sectional study
Source: Front Nutr. 2025 Apr 17;12:1529223. doi: 10.3389/fnut.2025.1529223 (PMC12043482; doi:10.3389/fnut.2025.1529223)

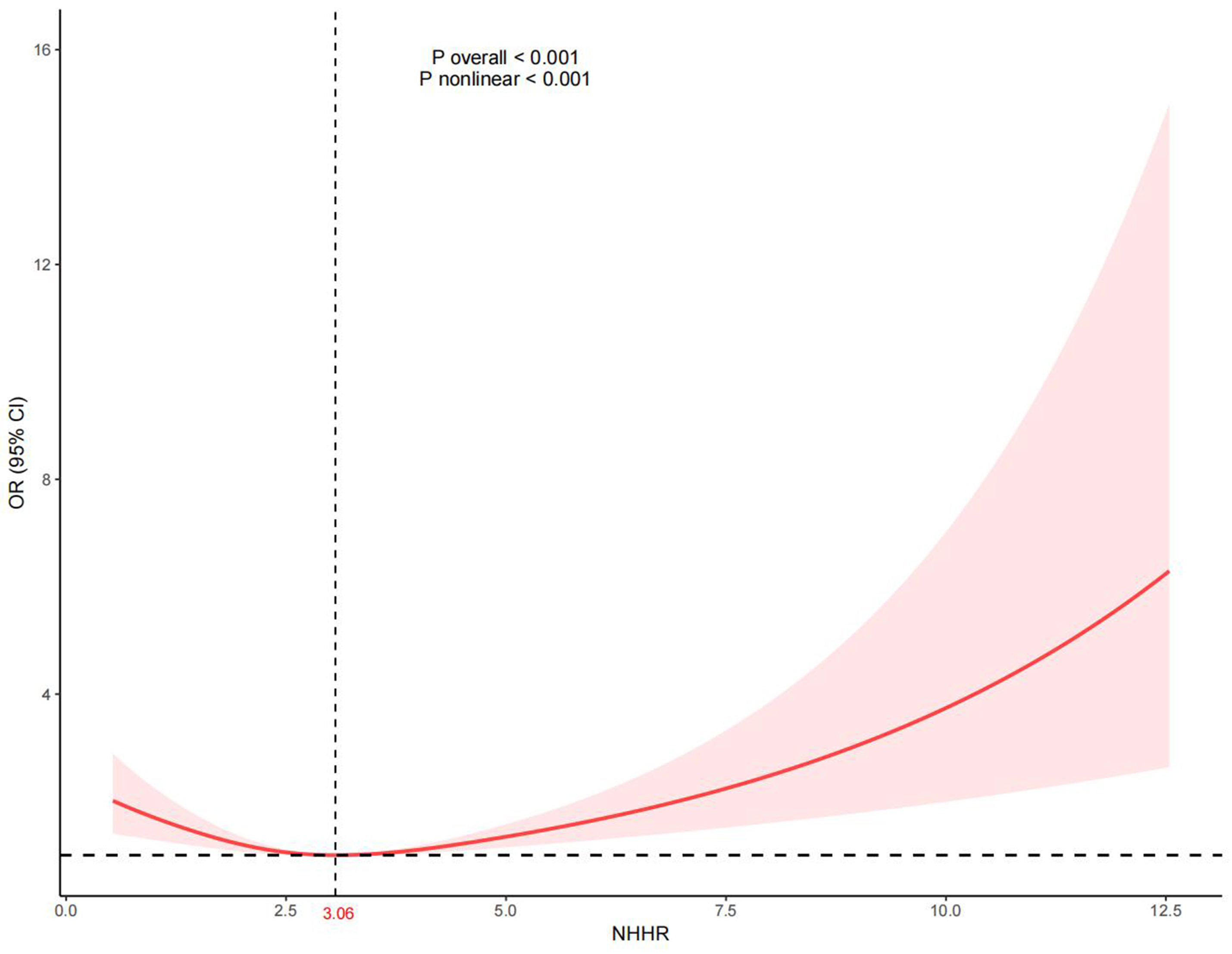

Supplement: Supplementary Figure S1 — RCS analysis of the association between NHHR and CVD after full adjustment, including LC9 as an additional. [file Image_1.jpg]

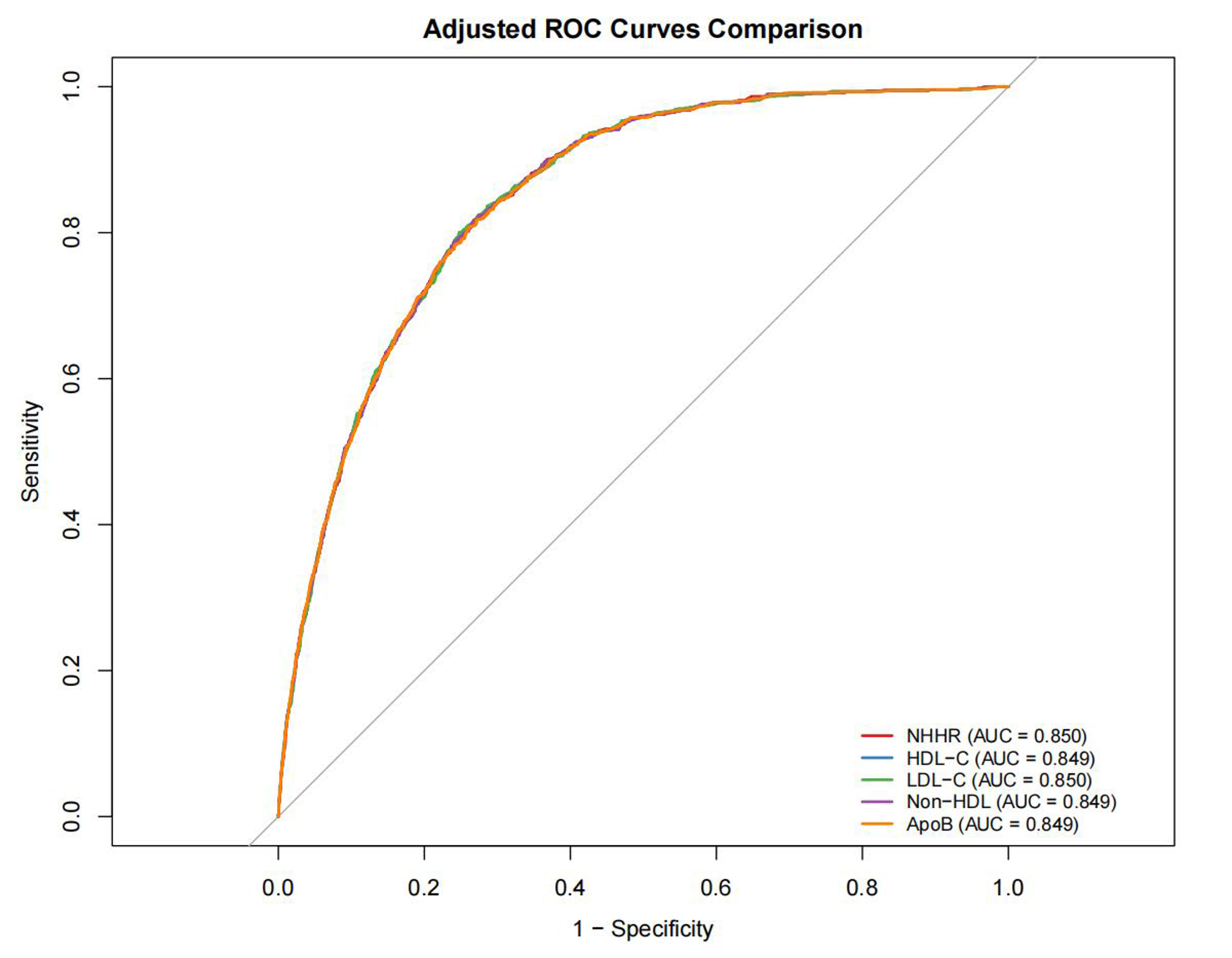

Supplement: Supplementary Figure S2 — ROC results. Comparison of NHHR with traditional lipid markers (LDL-C and apo B). [file Image_2.jpg]
