# Supplementary material for: Lipoprotein cholesterol ratios and cardiovascular disease risk in US adults: a cross-sectional study
Source: Front Nutr. 2025 Apr 17;12:1529223. doi: 10.3389/fnut.2025.1529223 (PMC12043482; doi:10.3389/fnut.2025.1529223)
Supplement: Supplementary Table S1 — The detail definition and classification of covariates. [file Table_1.docx]

| **Supplementary Table1. The detail definition and classification of covariates.** | |
| --- | --- |
|  | |
| **Variables** | **Definitions or Classification** |
| Sex | Male, Female. |
| Race | Non-Hispanic White, Non-Hispanic Black, Mexican American, Other Race. |
| Education attainment | High school or less, More than high school. |
| Marital status | Married or living with partner, Living alone. |
| PIR | Low (PIR ≤1), Middle (1< PIR < 4), and High (PIR ≥4). |
|  | Never smoking: <100 cigarettes in lifetime; |
| Smoking status | Former smoking: >100 cigarettes in life and smoke not at all now; |
|  | Now smoking: >100 cigarettes in lifetime. |
| Drinking status | Never: consumed fewer than 12 drinks in their lifetime; |
|  | Former: consumed at least 12 drinks in one year but did not drink in the last year or did not drink in the last year but had at least 12 drinks in their lifetime; |
|  | Heavy: consumed at least 3 drinks per day for females, at least 4 drinks per day for males, or engaged in binge drinking on 5 or more days per month; |
|  | Moderate: consumed at least 2 drinks per day for females, at least 3 drinks per day for males, or engaged in binge drinking on at least 2 days per month; |
|  | Mild : consumed at most 1 drink per day for females, at most 2 drinks per day for males. |
|  | An average systolic blood pressure (SBP) equal to or exceeding 140 mmHg; |
| Hypertension | An average diastolic blood pressure (DBP) equal to or exceeding 90 mmHg; |
|  | Self-reported hypertension; |
|  | Individuals taking prescribed anti-hypertensive medications. |
| CVD | The medical conditions section, identified by the variable name prefix MCQ, encompasses self- and proxy-reported personal interview data covering an extensive range of health conditions and medical history for both children and adults. This section includes inquiries such as ‘Has a doctor or other health professional ever informed you/SP that you/he/she… had congestive heart failure, coronary heart disease, angina (also called angina pectoris), heart attack (also called myocardial infarction), stroke, etc.?’ These questions, labeled as MCQ160B-F in the household questionnaires administered during home interviews, were utilized to identify participants with a history of CVD if they responded ‘yes’ to any of these questions. |
| BMI | weight (kg)/height (m^2^). |
| Laboratory tests | The Specific method can be found in this webpage |
|  | [(https://wwwn.cdc.gov/nchs/nhanes/continuousnhanes/labmethods.aspx?BeginYear=2003)](https://wwwn.cdc.gov/nchs/nhanes/continuousnhanes/labmethods.aspx?BeginYear=2017) |
| Abbreviations: T2DM, type 2 diabetes mellitus; PIR, family poverty income ratio; CVD, cardiovascular disease; TC, total cholesterol; TG, triglyceride; HDL-C, high-density lipoprotein cholesterol; LDL-C, low-density lipoprotein cholesterol; VAI, visceral adiposity index; BMI, body mass index. | |
