# Supplementary material for: Lipoprotein cholesterol ratios and cardiovascular disease risk in US adults: a cross-sectional study
Source: Front Nutr. 2025 Apr 17;12:1529223. doi: 10.3389/fnut.2025.1529223 (PMC12043482; doi:10.3389/fnut.2025.1529223)
Supplement: Supplementary Table S2 — Best threshold-based risk reclassification for NHHR, HDL-C, and non-HDL-C. [file Table_2.docx]

**Supplemental Table 2.**

| Variables | Best thresholds | Higher risk | Lower risk |
| --- | --- | --- | --- |
| NHHR | 3.05 | 4288 | 7183 |
| HDL-C | 1.32 | 5699 | 5772 |
| Non-HDL-C | 3.15 | 7471 | 4000 |
